# Supplementary figures and images for: Hypothesis generation for rare and undiagnosed diseases through clustering and classifying time-versioned biological ontologies
Source: PLoS One. 2024 Dec 26;19(12):e0309205. doi: 10.1371/journal.pone.0309205 (PMC11670971; doi:10.1371/journal.pone.0309205)

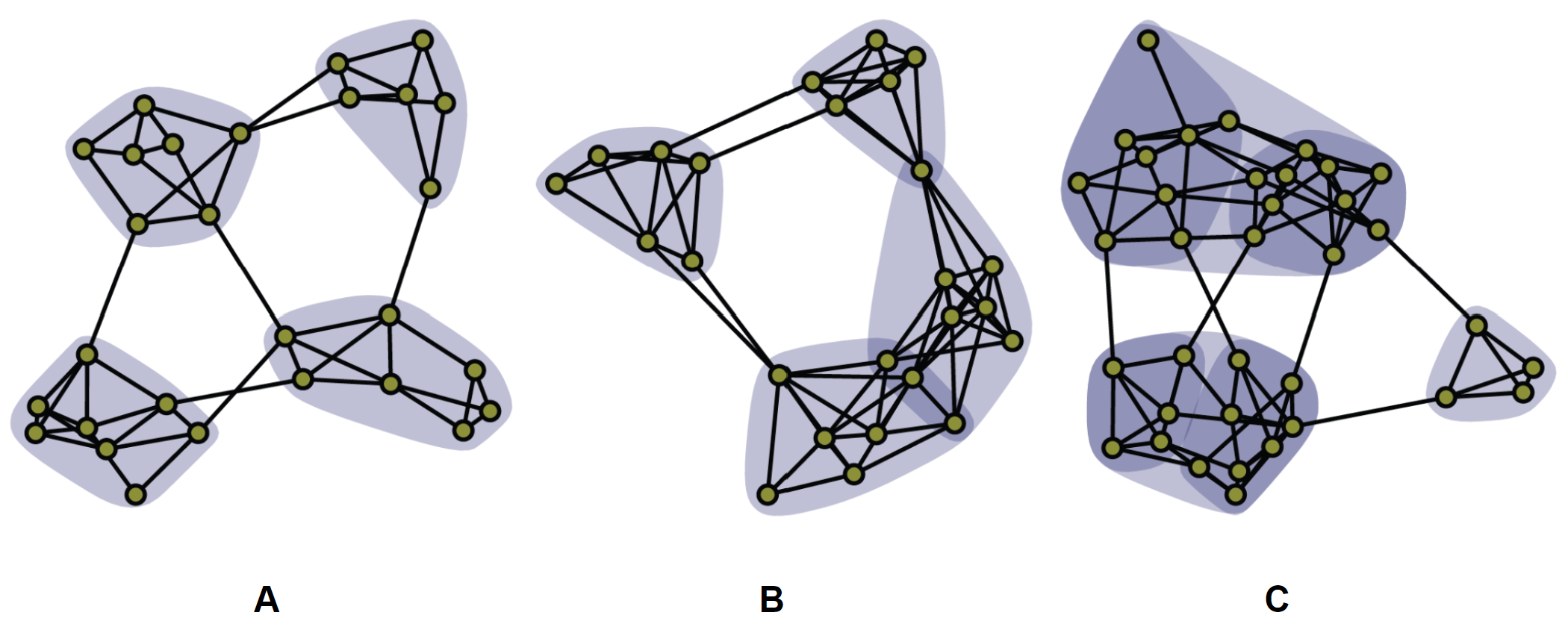

Supplement: S1 Fig — Adapted from [58]. (TIFF) [file pone.0309205.s001.tiff]

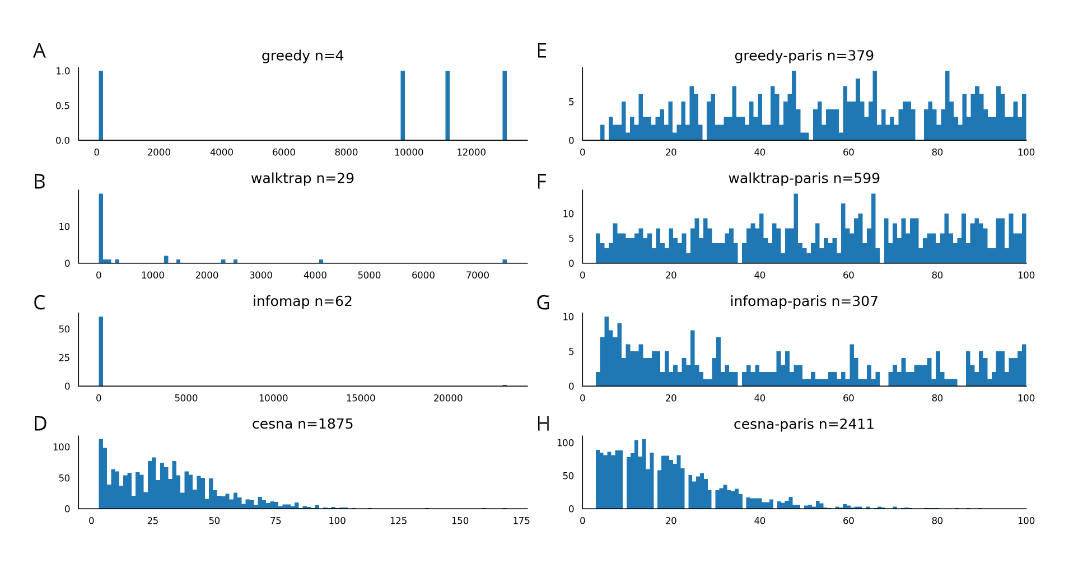

Supplement: S2 Fig — Shown are distributions of cluster size using A. greedy B. walktrap C. infomap and D. censa. The second column contains the subcluster size distributions, where every cluster from panels A-D were clustered again with the paris-hierarchical method E. greedy-paris F. walktrap-paris G. infomap-paris and H. censa-paris. In all of these plots the x-axis is cluster-size and the y-axis is the number of clusters. The first three of the clustering algorithms on their own, have a tendency to produce very few clusters that are all very large, some with a membership larger than 20,000 nodes, in the case of infomap. Our end goal is to use these clusters to provide sets of genes and phenotypes that are likely to have yet-to-be-discovered clinically meaningful relationships. These clusters in A-D are far too large to be useful for hypothesis generation in clinical or experimental settings. A second layer of cluster with the paris method is applied and shown in F-H, setting an upper limit of 100 on cluster size results in many more clusters of a size manageable for human curation. (TIFF) [file pone.0309205.s002.tiff]

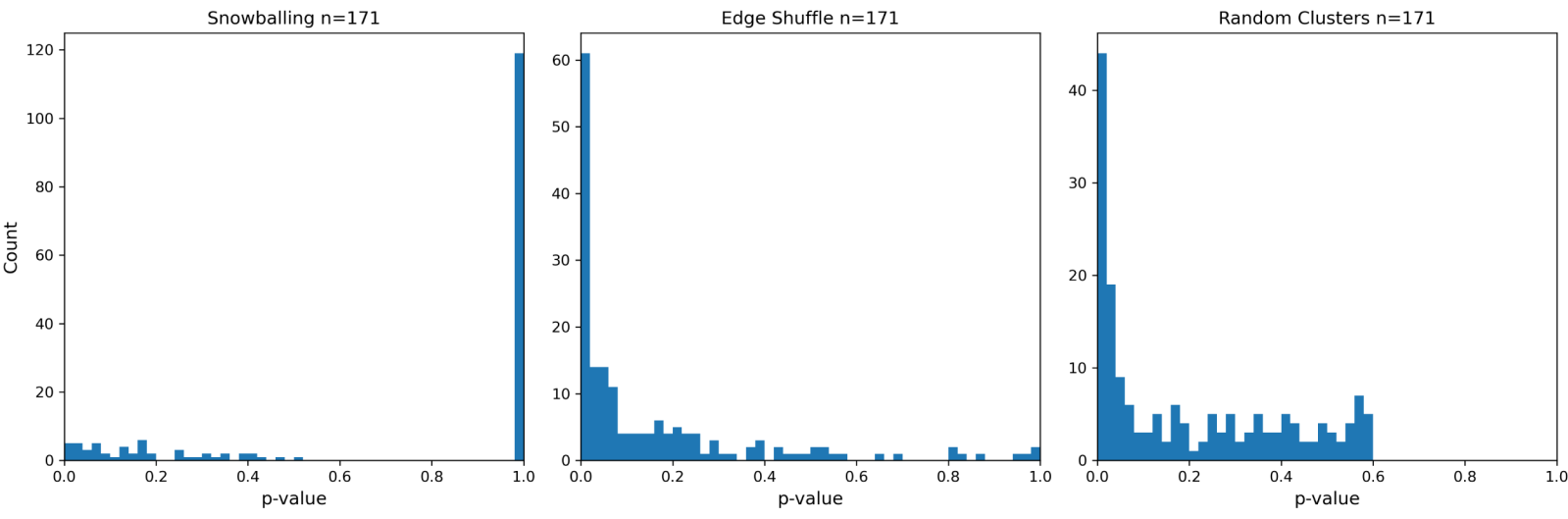

Supplement: S3 Fig — All figures share the same y-axis which is also on a log scale. The proportion of clusters with p < 0.05 in each model is 6%, 48%, and 39%, for snowballing, edge-shuffle, and random clusters respectively. Snowballing has 70% of its clusters with p = 1.00, whereas the other two models have zero clusters falling into this category. (PDF) [file pone.0309205.s003.pdf]
