# Supplementary material for: Hypothesis generation for rare and undiagnosed diseases through clustering and classifying time-versioned biological ontologies
Source: PLoS One. 2024 Dec 26;19(12):e0309205. doi: 10.1371/journal.pone.0309205 (PMC11670971; doi:10.1371/journal.pone.0309205)
Supplement: Attachment — Submitted filename: BOCC_rebuttal (1).pdf [file pone.0309205.s006.pdf]

Rebuttal Letter

The text in black is copied and pasted from the email from the editor. Our comments and response to each item is in red.

When submitting your revision, we need you to address these additional requirements.

1. Please ensure that your manuscript meets PLOS ONE's style requirements, including those for file naming. The PLOS ONE style templates can be found at https://journals.plos.org/plosone/s/file?id=wjVg/PLOSOne_formatting_sample_main_body.pdf and https://journals.plos.org/plosone/s/file?id=ba62/PLOSOne_formatting_sample_title_authors_affiliations.pdf

Updated formatting

2. Please note that PLOS ONE has specific guidelines on code sharing for submissions in which author-generated code underpins the findings in the manuscript. In these cases, all author-generated code must be made available without restrictions upon publication of the work. Please review our guidelines at https://journals.plos.org/plosone/s/materials-and-software-sharing#loc-sharing-code and ensure that your code is shared in a way that follows best practice and facilitates reproducibility and reuse

License added to github, Data Availability Statement updated

3. Please note that PLOS ONE has specific guidelines on code sharing for submissions in which author-generated code underpins the findings in the manuscript. In these cases, all author-generated code must be made available without restrictions upon publication of the work. Please review our guidelines at https://journals.plos.org/plosone/s/materials-and-software-sharing#loc-sharing-code and ensure that your code is shared in a way that follows best practice and facilitates reproducibility and reuse

Data Availability Statement section has been added with details about code and data access.

4. We suggest you thoroughly copyedit your manuscript for language usage, spelling, and grammar. If you do not know anyone who can help you do this, you may wish to consider employing a professional scientific editing service.

Whilst you may use any professional scientific editing service of your choice, PLOS has partnered with both American Journal Experts (AJE) and Editage to provide discounted services to PLOS authors. Both organizations have experience helping authors meet PLOS guidelines and can provide language editing, translation, manuscript formatting, and figure formatting to ensure your manuscript meets our submission guidelines. To take advantage of our partnership with AJE, visit the AJE website (http://learn.aje.com/plos/) for a 15% discount off AJE services. To take advantage of our partnership with Editage, visit the Editage website (http://www.editage.com) and enter referral code PLOSEDIT for a 15% discount off Editage services. If the PLOS editorial team finds any language issues in text that either AJE or Editage has edited, the service provider will re-edit the text for free.

Upon resubmission, please provide the following:
● The name of the colleague or the details of the professional service that edited your manuscript
● A copy of your manuscript showing your changes by either highlighting them or using track changes (uploaded as a *supporting information* file)
● A clean copy of the edited manuscript (uploaded as the new *manuscript* file)

Edits were made by Michael Bradshaw and Ryan Layer. Changes were tracked with Overleaf and will be provided.

5. Thank you for stating the following financial disclosure: "This work was supported by a grant from Children's Hospital Colorado."
Please state what role the funders took in the study. If the funders had no role, please state: ""The funders had no role in study design, data collection and analysis, decision to publish, or preparation of the manuscript.""
If this statement is not correct you must amend it as needed.
Please include this amended Role of Funder statement in your cover letter; we will change the online submission form on your behalf.

Elaborated members of Children’s Hospital were involved in this study saying:
This work was supported by a grant from Children's Hospital Colorado. Members of the funding body collected the patient data, aided in the direction of analysis, and are authors of the paper.

6. Thank you for stating the following in the Acknowledgments Section of your manuscript: "This work was supported by a grant from Children's Hospital Colorado."
We note that you have provided funding information that is not currently declared in your Funding Statement. However, funding information should not appear in the Acknowledgments section or other areas of your manuscript. We will only publish funding information present in the Funding Statement section of the online submission form.
Please remove any funding-related text from the manuscript and let us know how you would like to update your Funding Statement. Currently, your Funding Statement reads as follows: "This work was supported by a grant from Children's Hospital Colorado."

Please include your amended statements within your cover letter; we will change the online submission form on your behalf.

Amended text has been added to the cover letter.

7. We note that you have indicated that there are restrictions to data sharing for this study. For studies involving human research participant data or other sensitive data, we encourage authors to share de-identified or anonymized data. However, when data cannot be publicly shared for ethical reasons, we allow authors to make their data sets available upon request. For information on unacceptable data access restrictions, please see http://journals.plos.org/plosone/s/data-availability#loc-unacceptable-data-access-restrictions.

Before we proceed with your manuscript, please address the following prompts:

a) If there are ethical or legal restrictions on sharing a de-identified data set, please explain them in detail (e.g., data contain potentially identifying or sensitive patient information, data are owned by a third-party organization, etc.) and who has imposed them (e.g., a Research Ethics Committee or Institutional Review Board, etc.). Please also provide contact information for a data access committee, ethics committee, or other institutional body to which data requests may be sent.

b) If there are no restrictions, please upload the minimal anonymized data set necessary to replicate your study findings to a stable, public repository and provide us with the relevant URLs, DOIs, or accession numbers. Please see http://www.bmj.com/content/340/bmj.c181.long for guidelines on how to de-identify and prepare clinical data for publication. For a list of recommended repositories, please see https://journals.plos.org/plosone/s/recommended-repositories. You also have the option of uploading the data as Supporting Information files, but we would recommend depositing data directly to a data repository if possible.

Please update your Data Availability statement in the submission form accordingly.

Data Availability statement has been updated to include details about requesting access to CHCO patient records. The patient data from MyGene2, all of which is publicly available is included in S1_dataset.csv

8. Please include captions for your Supporting Information files at the end of your manuscript, and update any in-text citations to match accordingly. Please see our Supporting Information guidelines for more information: http://journals.plos.org/plosone/s/supporting-information.

Supporting info section added

9. We notice that your supplementary figures are uploaded with the file type 'Figure'. Please amend the file type to 'Supporting Information'. Please ensure that each Supporting Information file has a legend listed in the manuscript after the references list.

Supplementary figures have been properly labeled

10. Please review your reference list to ensure that it is complete and correct. If you have cited papers that have been retracted, please include the rationale for doing so in the manuscript text, or remove these references and replace them with relevant current references. Any changes to the reference list should be mentioned in the rebuttal letter that accompanies your revised manuscript. If you need to cite a retracted article, indicate the article's retracted status in the References list and also include a citation and full reference for the retraction notice.

Duplicate citations removed

Additional Editor Comments:

The main comments made by the reviewers on the manuscript about the Biological Ontology Cluster Classification (BOCC) tool can be summarized as follows:

1. Figures in the manuscript are not properly labeled.

Figures labels have been updated to match PLOS guidelines.

2. Inconsistency in font face and clarity between text and images.
3. Lack of clarity on the novelty of the study needs highlighting in the introduction.
4. Absence of a literature review section to establish context and credibility.

A literature review has been added to the introduction.

5. The manuscript seems outdated due to the limited citation of recent works; it needs updating with suggested references made by the reviewers, which I suggest you incorporate in your article, such as the introduction section or literature review section.

Additional references have been added in the literature review section.

6. Recommendations for proofreading to correct grammatical mistakes.

Proof reading has been done and edits made

7. Suggestion to compare results with state-of-the-art studies for validation.

We agree with you about the importance of comparing tools. Our manuscript includes comparisons with widely used clustering algorithms and null models. More specifically, we

compared four pre-existing and widely used clustering algorithms and a comparison of the traditional random-graph based null models vs the more conservative snow-ball sampling-based approach. To make this easier to find, we have added clearer language about our comparisons and findings. As for comparing high level results of BOCC to other tools, we do not know of any other method that unbiasedly discovers co-occurring genotype/phenotype pairs. While it is module identification tools and variant prioritization tools do yield pairs, their search is biases toward pathways or patients. If the reviewers are aware of tool directly comparable to BOCC that we are not, we will be happy to perform a comparison and cite them.

8. Absence of a discussion on the limitations of the study; recommended for inclusion in the conclusion.

We agree on the importance of a discussion of the limitations, discussion has been added.

9. Overall, the paper is well-written and suitable for the journal but lacks a conclusion section to summarize findings and implications.

Per PLOS ONE submission and formatting guidelines a conclusion section is not required and can be combined with the discussion and or results section. See https://journals.plos.org/plosone/s/submission-guidelines#loc-results-discussion-conclusions

“Results, Discussion, Conclusions
These sections may all be separate, or may be combined to create a mixed Results/Discussion section (commonly labeled “Results and Discussion”) or a mixed Discussion/Conclusions section (commonly labeled “Discussion”).”

We found for the sake of narrative integrating the discussion and conclusion seemed best. But we acknowledge that if the reviewers miss that information it should be clearer, so we have added emphasising language in the discussion section about we conclude.

This summary addresses the key areas the reviewers have identified for improvement, including technical presentation, content depth, literature context, and overall structure.

[Note: HTML markup is below. Please do not edit.]

Reviewers' comments:

Reviewer's Responses to Questions
Comments to the Author

1. Is the manuscript technically sound, and do the data support the conclusions?

The manuscript must describe a technically sound piece of scientific research with data that supports the conclusions. Experiments must have been conducted rigorously, with appropriate controls, replication, and sample sizes. The conclusions must be drawn appropriately based on the data presented.

Reviewer #1: Yes

Reviewer #2: Yes

Reviewer #3: Yes

2. Has the statistical analysis been performed appropriately and rigorously?

Reviewer #1: Yes

Reviewer #2: Yes

Reviewer #3: Yes

3. Have the authors made all data underlying the findings in their manuscript fully available?

The PLOS Data policy requires authors to make all data underlying the findings described in their manuscript fully available without restriction, with rare exception (please refer to the Data Availability Statement in the manuscript PDF file). The data should be provided as part of the manuscript or its supporting information, or deposited to a public repository. For example, in addition to summary statistics, the data points behind means, medians and variance measures should be available. If there are restrictions on publicly sharing data—e.g. participant privacy or use of data from a third party—those must be specified.

Reviewer #1: Yes

Reviewer #2: Yes

Reviewer #3: Yes

4. Is the manuscript presented in an intelligible fashion and written in standard English?

PLOS ONE does not copyedit accepted manuscripts, so the language in submitted articles must be clear, correct, and unambiguous. Any typographical or grammatical errors should be corrected at revision, so please note any specific errors here.

Reviewer #1: No

Reviewer #2: Yes

Reviewer #3: Yes

We and collegaes have done a general full proofreading of the manuscript to catch any errors.

5. Review Comments to the Author

Please use the space provided to explain your answers to the questions above. You may also include additional comments for the author, including concerns about dual publication, research ethics, or publication ethics. (Please upload your review as an attachment if it exceeds 20,000 characters)

Reviewer #1: the study presents an innovative tool named Biological Ontology Cluster Classification (BOCC). This tool is aimed at assisting in the diagnosis of rare and undiagnosed diseases by identifying potential gene-to-phenotype (g2p) associations that are not explicitly documented in the current literature. Here are my comments on the manuscript:

1. All the figures are not properly labeled.
Thank you for noting this, figure labels have been fixed.

2. the manuscript has different font face. for instance, I dont know how I will classify the text/image between line 50 and 51. Is it text or image, if it is text, while did it have different front face and if it is image while is it not label?

Formatting of this section has been edited to match PLOS's guidelines.

3. I understand that BOCC is available as both a web application and a command-line tool, making it accessible for different types of users ranging from researchers to clinicians. However, what is the novelty of this study? the author is expected to highlights the contributions of this study towards the last paragraph of the introduction section.

The novelty of BOCC has now been highlighted in the introduction, it was previously only discussed at length in the Discussion section.

4. I suggest that the author should create a section to discuss the literature review of some related work done in this area so as to enhance the credibility of this study.

A literature review has been added to the introduction, complete with a table!

6. Havn't checked this manuscript thoroughly, I observed that the author makes little efforts to cite and reference 2023 work. This act makes the manuscript looks outdated. Therefore, I will suggest that the author should make use of this following searched references to updated their manuscript:

Thank you for this suggestion to include more recent citations. We have included additional references in the literature review and discussion sections. We have carefully reviewed each paper listed below and comment on why we chose to cite it or not.

1. Zhuang, Y., Jiang, N., Xu, Y., Xiangjie, K., & Kong, X. (2022). Progressive Distributed and Parallel
Similarity Retrieval of Large CT Image Sequences in Mobile Telemedicine Networks. Wireless communications and mobile computing, 2022. doi: 10.1155/2022/6458350
We appreciate this article deals with human health and time series data. But discussing computer vision applications to CT scan is out of scope with our study.
2. Lu, G., Duan, L., Meng, S., Cai, P., Ding, S.,... Wang, X. (2023). Development of a colorimetric and turn-on fluorescent probe with large Stokes shift for H2S detection and its multiple applications in environmental, food analysis and biological imaging. Dyes and Pigments, 220, 111687. doi:
https://doi.org/10.1016/j.dyepig.2023.111687
This is an interesting development in tagging molecules. But, our manuscript does is focused on computation methods, not physical molecular binding or identification.
3. Siyu Lu, J. Y. B. Y. (2023). Analysis and Design of Surgical Instrument Localization Algorithm. Computer Modeling in Engineering & Sciences, 137(1), 669-685. doi:
10.32604/cmes.2023.027417
While more algorithmicly inclined, this study focused on physical localisation of surgical devices. Our study has nothing to do with surgery, or localization, which is out of scope for our study.
4. Zhu, Y., Huang, R., Wu, Z., Song, S., Cheng, L.,... Zhu, R. (2021). Deep learning-based predictiveidentification of neural stem cell differentiation. Nature Communications, 12(1), 2614. doi:
10.1038/s41467-021-22758-0
Like this study, ours also uses machine learning for biomedical purposes. But the feature based classification used in our manuscript are substantially different from the the deeplearning image recognition techniques used here.
5. Chen, L., He, Y., Zhu, J., Zhao, S., Qi, S., Chen, X.,... Xie, T. (2023). The roles and mechanism of
m6A RNA methylation regulators in cancer immunity. Biomedicine & Pharmacotherapy, 163, 114839. doi: https://doi.org/10.1016/j.biopha.2023.114839
Our manuscript deals with rare diseases, the underlying data we use is limited to genes and proteins abstractly represeted as nodes in a graph, we do not use methylations and RNA. While some specific cancers fall into the category of rare disease, they are a class of disease our method does not work on.

6. Huang, H., Liu, L., Wang, J., Zhou, Y., Hu, H., Ye, X.,... Tang, B. Z. (2022). Aggregation caused
quenching to aggregation induced emission transformation: a precise tuning based on BN-doped
polycyclic aromatic hydrocarbons toward subcellular organelle specific imaging. Chemical Science, 13(11), 3129-3139. doi: 10.1039/D2SC00380E

This paper is a biochemical study, where as ours is focused on learning and integrating biological networks.

7. Huang, H., Wu, N., Liang, Y., Peng, X., & Shu, J. (2022). SLNL: A novel method for gene selection
and phenotype classification. International Journal of Intelligent Systems, 37(9), 6283-6304. doi: https://doi.org/10.1002/int.22844
Thank you for bring this paper to our attention, we have include this citation in our discussion of future work.

5. I suggest that the manuscript should be thoroughly proof-read to avoid some grammar mistake.

We and collegaes have done a general full proofreading of the manuscript to catch any errors.

6. I recommend that the author should compare their result with the state-of-the-art studies so as to validate the strength of the obtained result.

We agree with you about the importance of comparing tools. Our manuscript includes comparisons with widely used clustering algorithms and null models. More specifically, we compared four pre-existing and widely used clustering algorithms and a comparison of the traditional random-graph based null models vs the more conservative snow-ball sampling-based approach. To make this easier to find, we have added clearer language about our comparisons and findings. As for comparing high level results of BOCC to other tools, we do not know of any other method that unbiasedly discovers co-occurring genotype/phenotype pairs. While it is module identification tools and variant prioritization tools do yield pairs, their search is biases toward pathways or patients. If the reviewers are aware of tool directly comparable to BOCC that we are not, we will be happy to perform a comparison and cite them.

7. What is the limitation of this study. This can be included in the conclusion section to give room for future research.

Discussion of the limitations, namely the biases the underlying networks bring with them has been added to the discussion.

Reviewer #2: This manuscript is well written, introducing their BOCC tool. As they write, BOCC is a series of network-science-based methodologies that identify relevant clusters from a heterogeneous network comprised of HPO, STRING, OMIM, and Orphanet. I do not have any major suggestions for how to improve this paper, and it seems suitable for this journal venue.

Thank you, we appreciate your comments.

Reviewer #3: The research addressed the topic of discuss but there are areas to be improved. The importance of literature review cannot be overemphasized. it is of great importance as it serves the purpose of establishing the context of a research study by elucidating the existing knowledge on the subject matter.This aids researchers in situating their work within the already established body of knowledge. Secondly, it allows researchers to identify gaps within the current knowledge base, which can subsequently form the foundation for research questions or hypotheses. Thirdly, a literature review supports the justification of research methodologies or methods by showcasing the manner in which prior studies were conducted and their contributions to the field.

We wholeheartedly agree, a literature review has been added.

Therefore as observed that this important section is missing in the work, I suggest that it be included and relevant work be added to improve this research. I suggest the following work:

Thank you for this suggestion to include more citations. We have included additional references in the literature review and discussion sections. We have carefully reviewed each paper listed below and comment on why we chose to cite it or not.

1. Luo, Y., Chen, D., & Xing, X. (2023). Comprehensive Analyses Revealed Eight Immune Related Signatures Correlated With Aberrant Methylations as Prognosis and Diagnosis Biomarkers for Kidney Renal Papillary Cell Carcinoma. Clinical Genitourinary Cancer, 21(5), 537-545. doi: https://doi.org/10.1016/j.clgc.2023.06.011

This paper are focused on improving diagnoses, this one focused on the identification of various biomarkers, methylation and expression and their relationship with a specific form of cancers. In our study we focus on not single disease, but rare and undiagosed diseases as a whole.

2. Gan, Y., Xu, Y., Zhang, X., Hu, H., Xiao, W., Yu, Z.,... Zheng, S. (2023). Revisiting Supersaturation of a Biopharmaceutical Classification System IIB Drug: Evaluation via a Multi-Cup Dissolution Approach and Molecular Dynamic Simulation. Molecules , 28(19), 6962. doi: https://doi.org/10.3390/molecules28196962

While biological networks are used in drug discover, our paper does not address drug discovery.

3. Fan, Z., He, Y., Sun, W., Li, Z., Ye, C.,... Wang, C. (2023). Clinical characteristics, diagnosis and management of Sweet syndrome induced by azathioprine. Clinical and Experimental Medicine, 23, 3581-3587. doi: 10.1007/s10238-023-01135-9

4. Wu, J., Fang, Z., Wang, X., Zeng, W., Zhao, Y., Jiang, F.,... Li, J. (2022). SLIT2 Rare Sequencing Variants Identified in Idiopathic Hypogonadotropic Hypogonadism. Hormone Research in Paediatrics, 95(4), 384-392. doi: 10.1159/000525769

5. Gong, T., Zhang, F., Feng, L., Zhu, X., Deng, D., Ran, T.,... Ji, X. (2023). Diagnosis and surgical outcomes of coarctation of the aorta in pediatric patients: a retrospective study. Frontiers in Cardiovascular Medicine, 10. doi: 10.3389/fcvm.2023.1078038

6. Fan, Z., He, Y., Sun, W., Li, Z., Ye, C., & Wang, C. (2023). Amoxicillin-induced aseptic meningitis: clinical features, diagnosis and management. European journal of medical research, 28(1), 301. https://doi.org/10.1186/s40001-023-01251-y

Papers 3,4,5 and 6 investigate the clinical characteristics of a single disease, our method is agnostic to any specific disease and instead study rare and undiagosed diseases as a whole.

7. Jin, K., Gao, Z., Jiang, X., Wang, Y., Ma, X., Li, Y.,... Ye, J. (2023). MSHF: A Multi-Source Heterogeneous Fundus (MSHF) Dataset for Image Quality Assessment. Scientific Data, 10(1), 286. doi: 10.1038/s41597-023-02188-x

This is a database of images that can be used for machine learning tasks, our study does not consider images.

The introduction should include wht the work is contribution to the body of knowledge and conclude with section of the work.

The conclusion furnishes a brief overview of the primary discoveries and outcomes of the investigation, highlighting the significant implications of the study. It permits the researcher to contemplate on the degree to which the study has accomplished its objectives and whether the research inquiries have been addressed. Implications: The conclusion provides an occasion to deliberate upon the ramifications of the research findings, encompassing their pertinence to the research domain, practical implementations, and prospective influence.
I observed that there is no conclusion. I suggest this section be included.

Per PLOS ONE submission and formatting guidelines a conclusion section is not required and can be combined with the discussion and or results section. See https://journals.plos.org/plosone/s/submission-guidelines#loc-results-discussion-conclusions

“Results, Discussion, Conclusions
These sections may all be separate, or may be combined to create a mixed Results/Discussion section (commonly labeled “Results and Discussion”) or a mixed Discussion/Conclusions section (commonly labeled “Discussion”).”

We found for the sake of narrative integrating the discussion and conclusion seemed best. But we acknowledge that if the reviewers miss that information it should be clearer, so we have added emphasising language in the discussion section about we conclude.

6. PLOS authors have the option to publish the peer review history of their article (what does this mean?). If published, this will include your full peer review and any attached files.

If you choose “no”, your identity will remain anonymous but your review may still be made public.

Do you want your identity to be public for this peer review? For information about this choice, including consent withdrawal, please see our Privacy Policy.

Reviewer #1: No

Reviewer #2: No

Reviewer #3: No

[NOTE: If reviewer comments were submitted as an attachment file, they will be attached to this email and accessible via the submission site. Please log into your account, locate the manuscript record, and check for the action link "View Attachments". If this link does not appear, there are no attachment files.]

While revising your submission, please upload your figure files to the Preflight Analysis and Conversion Engine (PACE) digital diagnostic tool, https://pacev2.apexcovantage.com/. PACE helps ensure that figures meet PLOS requirements. To use PACE, you must first register as a user. Registration is free. Then, login and navigate to the UPLOAD tab, where you will find detailed instructions on how to use the tool. If you encounter any issues or have any questions when using PACE, please email PLOS at figures@plos.org. Please note that Supporting Information files do not need this step.
