# Supplementary material for: Hypothesis generation for rare and undiagnosed diseases through clustering and classifying time-versioned biological ontologies
Source: PLoS One. 2024 Dec 26;19(12):e0309205. doi: 10.1371/journal.pone.0309205 (PMC11670971; doi:10.1371/journal.pone.0309205)
Supplement: Attachment — Submitted filename: response_to_reviewers (1).pdf [file pone.0309205.s007.pdf]

Response to Reviewers

Major comments:
1. The formatting and language are usually relatively minor during review. However, multiple reviewers have already pointed it out in the first review. Several grammatical errors and formatting errors persist in the manuscript. I think the author should take it very seriously. I still observed many formatting issues, such as a missing space before the citation. For example, in page 2, “Matchmaker Exchange[2] and MyGene2[1]”. Many issues might be easily detected by Grammarly or other tools. I will recommend really finding an English editing service for the proofreading.

We apologize for the mistakes, thank you for bringing them to our attention. We have used grammarly and have had multiple native English speakers proofread and edit the manuscript for this revision. We hope you find it satisfactory.

2. In page 2, these two sentences: “Using the Human Phenotype Ontology, we can expand the patient’s assigned phenotypes to include all closely related phenotypes and diseases. By connecting STRING and HPO [4] with the gene-to-phenotype (g2p) connections from Orphanet[5] and OMIM[6], we can then look for indirect associations between the patient’s assigned data points.”
HPO was not introduce as abbriveation after the first appearance. And the citation for HPO should be after the first appearance.

Thank you for your attention to detail; we have fixed this oversight.

3. Page 3: “There is a trend toward using an ensemble of methods or methods capable of using higher-order patterns and integrating multiple types of networks (Table 1).” Redundant space before “)”.

This has been addressed, thank you.

4. Page 10: “Fig 4. A. Procedure for training and evaluating the XGboost model. Features were generated about each cluster (identified as described in Section)”, which Section? Is the Section number missing here? In page 11: the same issue, “After performing quality control (Section), there were on average 401 variants per patient (Fig 6.C).”

Missing section numbers have been added, thank you.

5. The author refers g2p to gene to phenotype. Does the author use Phenotype in this paper as a feature (HPO term)? We often use phenotype to refer to disorders and describe HPO terms by features.

No, we do not use HPO terms directly as features. HPO terms can be used as features - that is a valid methodological approach; but that is not the approach used here.

6. The author's definition of the undiagnosed patient in CHCO and MyGene2 is unclear. The first sentence of the result section states, “We applied BOCC to 721 patients from Children’s Hospital Colorado (CHCO) with suspected genetic drivers of their diseases.” Does that mean these patients are undiagnosed?

Thank you for raising this point of confusion. We do not claim that all 721 CHCO patients are still undiagnosed, as we are not privy to that aspect of the clinical records nor do we have IRB approval to access that information. But we do know

1. They were suspected of having a disease of genetic origin - which is why they underwent WES sequencing
2. There are no known connections between their VUS and the HPO terms assigned by their doctor - a hallmark of an undiagnosed disease case as set forth by the Clinical validity requirement of the CDC’s ACCE model.

We have added clarifying language around this point in a paragraph starting at line 507.

7. Moreover, in MyGene2 section, “We found that presently 111 of these profiles contained no direct connection between any of their genes and any of their HPO terms; we assumed these cases to still be undiagnosed.”, I think it is problematic. It is dangerous to assume the patient is undiagnosed because there is no connection between HPO and the gene. I randomly checked some patients in Table S1 and Table 5. I found some of the patients are already published in the literature, and I believe they are diagnosed. The first question is the patient with ID 3071 (https://mygene2.org/MyGene2/familyprofile/3071/profile). It is the first patient in Table S1. This patient was already published in a paper with PMID (25142838) and annotated with Kabuki and KMT2D. Does the author consider this patient as diagnosed or undiagnosed? For example, patient ID 877 (https://mygene2.org/MyGene2/familyprofile/877/genetic/gene) is already published in https://www.ncbi.nlm.nih.gov/pmc/articles/PMC5082428/. This patient was also annotated with Joubert syndrome and a likely pathogenic mutation in B9D2. Besides, patient ID 1292 has likely pathogenic mutation in TNNT3 and was diagnosed as “Arthrogryposis, Distal, Type 1A”. And the author reported “Arthrogryposis multiplex congenita” as a new relationship to TNNT3. However, we can see from the disease name linked to TNNT3, which is “Arthrogryposis, distal, type 2B2,” and the entry in omim was updated in 2019. I would say this new relationship between this HPO and TNNT3 is not surprised at all because you can see it from the disease name. Therefore, I wonder what the meaning of this prediction is.

The fact that some of these MyGene2 cases have been solved and published is a vote of confidence for our tool; it shows that our predictions were correct! The bigger question is, if these cases are solved, is there no recorded association between them and the rare disease databases on which the edges in HPO are based? That is a question for the database and ontology curators.

We conceded that our assumption of undiagnosed in MyGene2 cases has limitations and have added a discussion of the points raised by reviewer 5 to the manuscript, see the new paragraph starting at line 507.

The reviewer has struck upon several outstanding issues, limitations but also intentional features of phenotype-based methods and biological ontologies more broadly:

Distinct meaning - "Arthrogryposis, Distal, Type 1A" is a different disease than "Arthrogryposis, distal, type 2B2". So while it may not be surprising that mutations to the same gene cause them, it would still be a new connection.
Specificity of terms/incompleteness of data - "Arthrogryposis multiplex congenita" is a leaf node in HPO - it is the most specific identifier currently available. Having more general terms like this one is beneficial in that it allows inferences to be made about a class phenotypes more broadly (like what is happening to patient 1292) or to catch cases where more precise terms do not exist and where terms of greater detail need to be added.

We have added some discussion about this topic at line 497, which highlights the need to continue expanding and improving these ontologies.

8. My main concern is that I don't know how I validate wheter the connection found by the cluster is correct or not. The experiement should start from validate on the existing relationship from diagnosed patients. Then we know that the relationship found in the cluster do have some meanings. Then, we might select some patients with a disease gene found after the model is built. For example, the model trained on the data from 2019, then we can check whether the model can predict the relationship between the HPO and a Gene X, which was found as a disease-causing gene in 2020. As pointed out previously, when the definition of diagnosed and undiagnosed is unclear, it is very difficult to validate whether the cluster is meaningful or not, even though the experiments reported many significant p-values from different methods.

Yes, what you described is exactly how our validation experiments work. We identify clusters on the 2019 data, then label those clusters according to if new edges in the 2020 graph are found with in the 2019 clusters. These clusters and labels are then used to train our machine learning classifiers which are then tested and evaluated on the 2021 data - showing that the procedure works. Unfortunately, we are unaware of a set of patient profiles that would enable such an experiment; but we are open to suggestions.

9. In the end, how the clinicians use this tool is unclear. There are many nodes in the graph, and many clusters were reported after the HPO and genes are given as input. It will be great to show more examples and the steps how the user should use. For example, I tried several HPOs linked to Cornelia de Lange syndrome (NIPBL gene), Long eyelashes (HP:0000527). Synophrys

(HP:0000664) and Highly arched eyebrow (HP:0002553). However, I only found NIPBL is the third claster. I believe it is the key features for CdLS (NIPBL). Therefore, how do I interpret the first two clusters without NIPBL? The experiment and results showed that this method is working, statistically. However, how to interpret the results and validate the clinical meaning is unclear.

Thank you for taking the time to use our tool! Since there are already known connections between these HPO terms and gene, our tool is of limited utility as it is intended to generate hypotheses about latent gene-to-phenotype connections, regardless we hope you enjoyed the visual interface.

It is odd NIPBL does not show up until cluster 3 for you. When we searched for all those HPO terms and NIPBL, it is present in the first cluster (see screenshot below). As for the interpretation of the first two clusters not having the gene in them, this is likely an artifact of the sorting procedure in the web interface. It sorts by the number of search term matches, then by the predicted p-value, and then breaks ties by sorting the cluster IDs alphabetically.

We have added additional instructions of the web app use at line 336; thank you for the recommendation.

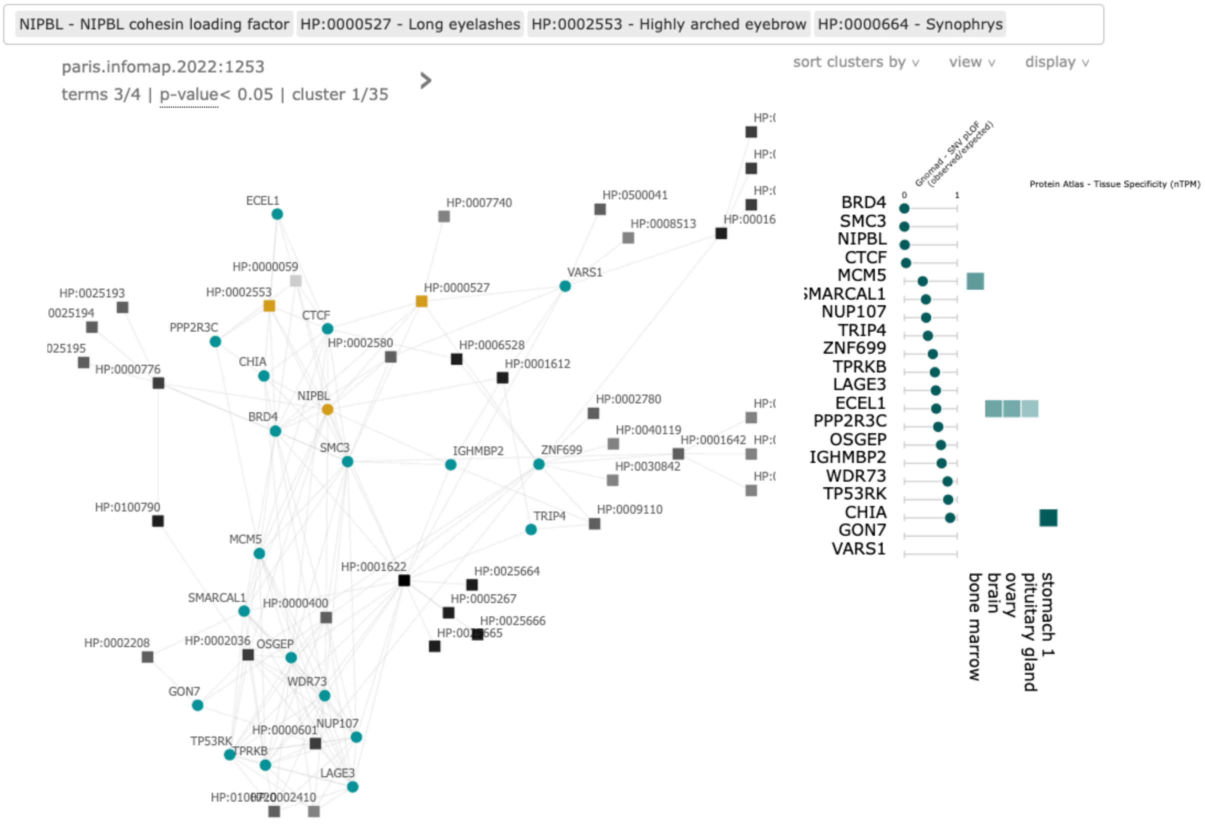

10. Will the gene in the same pathway or same phenotypic series shown in the same cluster?

Excellent question. Sometimes, but not always. Clustering algorithms are often used for identifying/ inferring genetic pathways and “disease modules” - or groups of closely related diseases. This was part of the motivation for using clustering. We this is mentioned in the introduction, but we have added text highlighting this later on at line 484.

11. In author’s first example, Patient 1930 with VUS in NBEA and SSPO. The author stated in the introduction that with more experiment to the associated phenotype in the clusters could help us to diagnose this patient. I believe the phenotype here is the HPO term. I wonder why we can use the new gene-to-hpo to solve the case with VUS? Does it contribute to the ACMG variant classification?

Nice observations. Yes, the phenotype and the HPO term are the same; we consider these terminologies more or less equivalent the difference being that HPO terms are precise and discrete and may not fulling encompass or describe all phenotypes. A predicted potential link between an HPO term and a VUS does not constitute a solved case; it is a promising hypothesis to be tracked down with further experimentation, literature, and or database research inorder to satisfy ACMG criteria. Our language around this was admittedly imprecise and has been updated, lines 54.
